# Unusual case of classic testicular seminoma in a 90-year-old patient: a case report

**DOI:** 10.1186/s13256-020-02517-3

**Published:** 2020-10-09

**Authors:** Ahmad Al-Mousa, Mohammad Nour Shashaa, Mohamad Shadi Alkarrash, Mohamad Alkhamis, Lina Ghabreau, Mouhsen Dandal

**Affiliations:** 1 Department of Urology, Faculty of Medicine, University of Aleppo, Aleppo, Syria; 2 Faculty of Medicine, University of Aleppo, Aleppo, Syria; 3 Department of Pathology, Faculty of Medicine, University of Aleppo, Aleppo, Syria

**Keywords:** Seminoma, Testicular tumor, Scrotum

## Abstract

**Background:**

Seminoma is the most common subtype of testicular cancer and occurs most commonly in patients aged 30–49 years, but decreases to a very low level in men in their 60s or older.

**Case presentation:**

A 90-year-old Syrian man with a 6-year history of an increase in size of his right scrotum, presented to the urological clinic and, on clinical examination, the findings suggested testicular tumor. After orchiectomy and histology results based on microscopic and immunohistochemical examinations, a pure seminoma was diagnosed, so we describe in this case report the second-oldest patient with classical seminoma in the medical literature.

**Conclusion:**

This case report has been written to focus on the probability of any type of testicular tumor occurring at any age or decade; urologists should consider seminoma as a differential diagnosis with any testicular swelling even in elderly patients.

## Background

Testicular cancers account for approximately 1–1.5% of all cancers in men and 5% of urological tumors [1]. It commonly presents as a painless, nodular unilateral mass [2].

Testicular cancers include germ cell tumors (GCTs) 90–95% and non-germ cells tumors (NGCTs)10–15%. GCTs are mainly categorized into unclassified type; seminoma (pure or classic, syncytiotrophoblastic and spermatocytic), and non-seminomatous-type tumors include embryonal carcinoma; yolk sac tumor; choriocarcinoma; other trophoblastic tumors; teratoma (mature, immature, or malignant); and mixed tumors [3, 4].

Seminoma is the most common testicular germ cell neoplasm and accounts for about 50% of all GCTs, which occur most commonly in patients aged 30–49 years [5].

Less than 20% of patients with stage I seminoma will have retroperitoneal lymph node metastatic disease [6].

In this case report, we describe a classic seminoma that was diagnosed in a 90-year-old patient, who is the second-oldest patient with this condition in the medical literature.

## Case presentation

A 90-year-old Syrian man with a 6-year history of an increase in size of his right scrotum, without pain presented to the urology clinic with enlargement of, and slight pain in, his right testis. He is a farmer with seven sons and has been smoking for 55 years. In his medical history, he had controlled hypertension and no history of testicular trauma or inflammation. A clinical examination showed the presence a painless, massive enlargement occupying his right scrotum, impermeable to light and with no inguinal lymph node palpable. His vital signs were within normal limits. A scrotal ultrasound scan showed a hypoechoic solid mass measuring approximately 10 cm in his right testis. Laboratory test results revealed tumor markers: alpha fetoprotein (AFP) of 3.1 KU/L (normal range 0.5–5.8 KU/L), the beta subunit of human chorionic gonadotropin (βhCG) of 2.38 IU/L (normal range 0–2.5 IU/L) and lactate dehydrogenase (LDH) of 420 IU/L (normal range 200–400 IU/L).

**Fig. 1:**
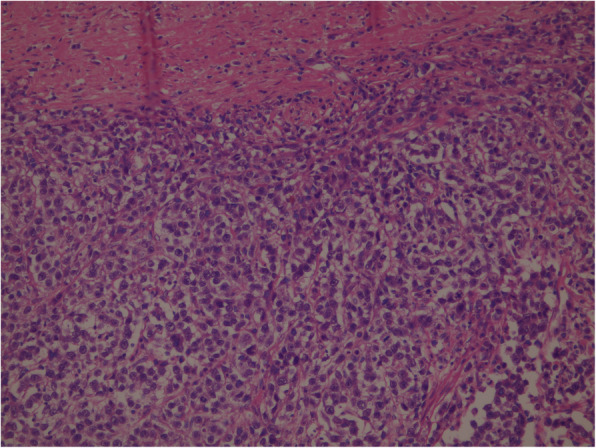
Sheets of polyhedral seminoma cells with a distinct cell membrane and large nuclei containing prominent nucleoli

**Fig. 2:**
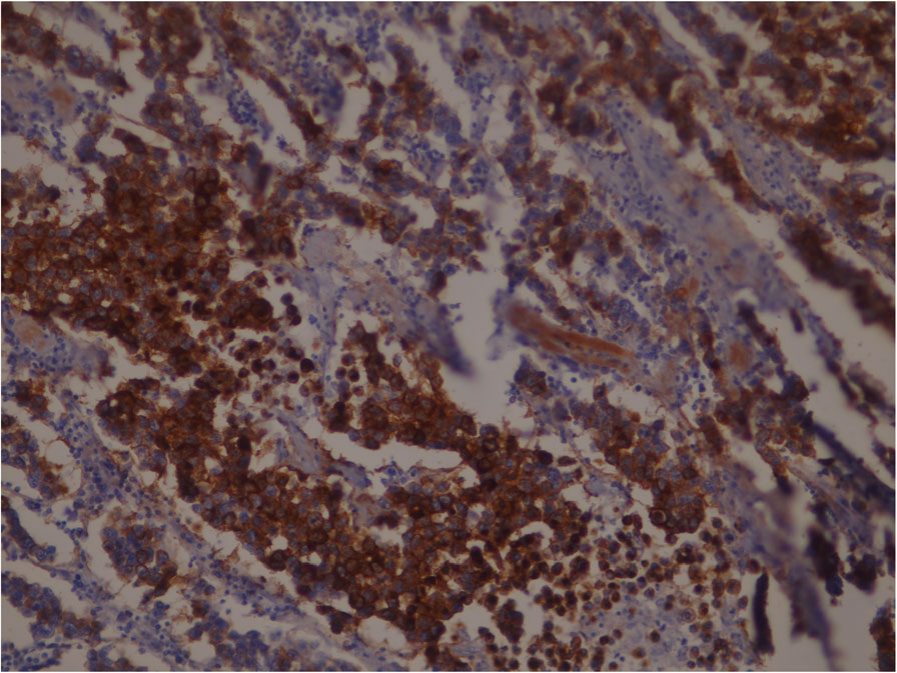
CD117(c-Kit) immunohistochemical stain that showing strong membranous positivity in seminoma cells

Routine blood test results were within normal limits. The findings suggested testicular tumor and a computed tomography (CT) scan of his abdomen and pelvis was performed and showed no retroperitoneum metastases. His right testicle was removed radically by a right inguinal approach. The pathology test result showed a tumor that is very rare in elderly patients, a classical testicular seminoma. A histological study revealed a nodular solid mass measuring 95*80 mm, with a solid gray cut surface with focal necrosis friable in consistency. The tumor had invaded the hilar soft tissues and tunica albuginea, but without tunica vaginalis invasion. Consequently, the stage was pT2, which it is when the tumor is limited to the testis (including rete testis invasion) with lymphovascular invasion or when the tumor invades the hilar soft tissue or epididymis or penetrates the visceral mesothelial layer covering the external surface of tunica albuginea, with or without lymphovascular invasion. Histological sectioning showed sheets of large polygonal cells (Fig. 1), which stained positively for CD117 (Fig. 2) and negative for cytokeratin and vimentin, which is consistent with a diagnosis of classical seminoma. A follow-up oncology consultation suggested outpatient surveillance without adjuvant chemotherapy. Our patient was hospitalized for 24 hours and discharged without complications. Tumor markers, hematological tests, and a CT evaluation were repeated after 6 months and again after 12 months, and all were within normal limits without any evidence for retroperitoneal metastases.

## Discussion and conclusion

Malignant tumors in the testis are rare. They include GCTs (seminoma and non-seminomatous-type tumors), which account for 90–95% of all primary testicular tumors, and sex cord-stromal tumors (Leydig cell, Sertoli cell, and gonadoblastoma) [7].

Classic testicular seminoma and non-seminoma tumors are not common in patients older than 60 years, while spermatocytic seminoma, malignant Leydig cell tumors and lymphomas, in addition to paratesticular sarcomas are more common among elderly patients [8].

The oldest case in the literature is a 92-year-old patient with classic seminoma [9].

Consequently, our patient is considered the second-oldest age in the literature, demonstrating that there is no age limit for this type of malignancy.

The patient often presents with a painless, slow-growing enlargement. Acute pain in the testis is noticed in about 10% of all cases and may be due to intratesticular hemorrhage or infraction, and in most cases a testicular mass is noticed. The mass is hard and the skin should be easily separable from it and testicular tumor may be accompanied by or covered with a hydrocele [7, 10].

Transillumination of the scrotum should be performed to distinguish between them and palpation of the abdomen may reveal a bulky retroperitoneal mass. Examination of supraclavicular, inguinal, and scalene nodes should be done also to detect any enlargement [7].

Serum AFP levels are not significantly elevated in patients with seminoma; one study reported very modest elevations in AFP levels in patients with histologically pure seminomas who had a typical clinical course. Serum hCG is often increased in the 10–20% of tumors with admixed syncytiotrophoblast cells. These levels are usually not in excess of 1000 mL U/mL, although one very large seminoma with abundant syncytiotrophoblasts had an associated hCG level of 4000 mL U/mL. Serum LDH is increased in about 80% of patients with advanced-stage disease [5].

Scrotal ultrasonography should be performed rapidly to assess for testicular tumor and to distinguish it from epididymal pathology. This technique may help to check for testicular tumor in the presence of a hydrocele [7].

CT scans of the abdomen and pelvis are used to assess for metastatic disease, especially in the lungs and retroperitoneum [7].

Seminoma is very radiosensitive and most of stage II seminomas are cured with radical orchiectomy and radiotherapy for retroperitoneal lymph nodes, while patients with advanced seminoma should receive primary chemotherapy. Seminomas are sensitive to platinum-based courses and 90% of patients with advanced disease respond to chemotherapy. Most of the residual retroperitoneal masses following chemotherapy are often fibrosis [11].

A positron emission tomography scan should be performed in a patient with a residual mass and, if positive, surgical resection is warranted [7].

In general, testicular tumors should be regularly followed up every 6 months for the first 3 years. During the follow-up, a careful examination of the other testis, the abdomen, and lymph nodes should be done and laboratory investigations, including AFP, hCG, and LDH levels, carried out.

Chest X-ray and an abdominopelvic CT scan are used less frequently to detect the risk of relapse in the retroperitoneum [12].

We describe in this case report the second-oldest patient (at 90 years old) diagnosed with classic seminoma in the medical literature. Therefore, classic seminoma could present at any age and should be considered as a differential diagnosis for any painless mass in the scrotum.

## Data Availability

The datasets used and/or analyzed in the current study is available from the corresponding author on reasonable request.

## Abbreviations

AFP: Alpha fetoprotein
βhCG: Beta subunit of human chorionic gonadotropin
CT: Computed tomography
GCT: Germ cell tumor
LDH: Lactate dehydrogenase
NGCT: Non-germ cells tumor
